# Reliability and Repeatability of Diffusion Tensor Imaging in Healthy and Pathological Patellar Tendons

**DOI:** 10.1002/jor.70156

**Published:** 2026-01-29

**Authors:** Elizabeth A. Schmida, Ethan Hansen, Daniel E. O'Brien, Diego Hernando, Kenneth S. Lee, Bryan C. Heiderscheit, Samuel A. Hurley, Naoaki Ito

**Affiliations:** ^1^ Department of Orthopedics and Rehabilitation University of Wisconsin‐Madison Madison Wisconsin USA; ^2^ Badger Athletic Performance Program University of Wisconsin‐Madison Madison Wisconsin USA; ^3^ Department of Radiology University of Wisconsin‐Madison Madison Wisconsin USA

**Keywords:** anterior cruciate ligament, microstructure, rehabilitation, tendinopathy

## Abstract

Patellar tendinopathy and bone‐patellar tendon‐bone autograft harvest for anterior cruciate ligament reconstruction are tendon injuries that impact long‐term knee health. Diffusion tensor imaging (DTI) is a non‐invasive magnetic resonance imaging (MRI) based approach with the potential to assess tendon microstructure. This study aimed to determine the inter‐rater reliability of segmentations and test‐retest repeatability of DTI metrics in pathological and contralateral patellar tendons. Ten participants received two bilateral knee MRI scans within a 7‐day period. 3D CUBE proton density weighted images and DTI were acquired. Two raters segmented each of the first scans, and one rater segmented the second scans. Tendon masks were then bisected into proximal and distal regions of equal length and trisected into medial, lateral, and central regions of equal width. From the DTI acquisition, diffusivities and fractional anisotropy averages were extracted. Intraclass correlations (ICCs) for inter‐rater reliability and test‐retest repeatability were calculated for each metric separated by limb (pathological vs contralateral tendon). Excellent inter‐rater reliability was observed for all DTI scalar metrics in all regions (ICCs from 0.920 to 0.994). Repeatability was poor to moderate in pathological tendons (0.164 to 0.709) and moderate to good in contralateral tendons (0.566 to 0.842).

**Statement of Clinical Significance:** While clinical implications of altered DTI scalar metrics in pathological tendons require further investigation, findings from this study provide clinicians and researchers with a reliable method for capturing patellar tendon microstructure.

## 1 Introduction

Patellar tendon injuries, including gradual onset tendinopathy [1] and iatrogenic injuries from bone‐patellar tendon‐bone autograft (BPTB) harvest for anterior cruciate ligament reconstruction (ACLR) [2, 3], are painful and impact long‐term knee health [4, 5]. Patellar tendinopathy presents in up to 25% of the active population [4] and can impair knee joint function [6, 7], physical activity levels [8], and quality of life [9]. Persistent anterior knee pain has been reported by 46% of patients [10] after ACLR, along with prolonged quadricep weakness [10, 11, 12, 13], and elevated rates of post‐traumatic knee osteoarthritis [14]. At the macrostructural level, focal tendon thickening and poor tissue quality in the medial third of the tendon are common characteristics in patellar tendinopathy [15, 16, 17]. Similarly, BPTB graft site tendons present with thickening across the tendon and particularly in the central third where the graft was harvested [11, 18, 19, 20].

While current clinical standard imaging modalities such as ultrasound imaging or magnetic resonance imaging (MRI) captures gross patellar tendon pathology (i.e., focal thickening, alterations to echogenicity or signal intensity) at the macrostructural level, these alterations do not always align with clinical presentation [21]. For instance, symptomatic improvements often precede the macrostructural healing seen on imaging in tendinopathic tendons and at the BPTB graft sites [22, 23]. These discrepancies suggest that current imaging modalities assessing tendon macrostructure may not provide a complete picture of the structural changes that occur in pathological tendons. At the microstructure level, studies have demonstrated pathological tendons consist of loosely packed, poorly organized collagen fibers [15], while healthy tendons consist of densely packed, parallel collagen fibers. These microstructure assessments, however, often require invasive procedures such as biopsies that are not clinically feasible [24, 25, 26].

Diffusion tensor imaging (DTI) is a non‐invasive MRI‐based approach with the potential for assessing tendon microstructural alterations in‐vivo. For standard DTI diffusion times (~50 ms), water molecules move distances of approximately 15 µm through tissue [27]. By quantifying water molecule diffusivity, DTI is sensitive to microstructural features (such as the organizational structure of collagen) on the order of 10 µm, allowing for microstructural quantification in tendons independent of voxel sizes that are typically much larger [28, 29]. DTI has shown promise for its application in tendons, by capturing microstructural differences in Achilles tendons with and without tendinopathy [30]. Findings from DTI in pathological patellar tendons may provide clinicians and researchers a more detailed understanding of patellar tendon pathophysiology and its clinical relevance than macrostructural observations alone.

To date, only non‐invasive in‐vivo assessments of healthy patellar tendon microstructure using DTI have been tested [31]. This study aimed to determine the reliability of DTI scalar metrics obtained from patellar tendon segmentations from separate raters and the repeatability of measuring DTI scalar metrics in pathological and contralateral patellar tendons on separate days.

## 2 Methods

### 2.1 Participants

This is a cross‐sectional cohort study (Level 3). Participants 18 years and older were recruited from sports medicine, rehabilitation, and sports recreation centers. For inclusion, participants with patellar tendinopathy must have presented with symptoms of tendinopathy as defined by the International Scientific Tendinopathy Symposium Consensus on Clinical Terminology (ICON) statement [1] along with macromorphological alterations (i.e., focal thickening or altered echogenicity) confirmed on B‐mode ultrasound imaging performed by a physical therapist with 6 years of musculoskeletal ultrasound experience. Participants with a BPTB graft harvest must have had an ACLR using an ipsilateral BPTB graft and been cleared to perform maximum effort hop, jump, and strength testing. Evidence of BPTB graft harvest was confirmed via ultrasound imaging. Participants with a history of invasive procedures to the patellar tendon (aside from a BPTB autograft harvest) such as platelet‐rich‐plasma injections, tenotomies, or tendon repairs were excluded. Participants provided written informed consent, and the study was approved by the Institutional Review Board at the University of Wisconsin‐Madison. Participants completed the Victorian Institute of Sports Assessment—Patellar Tendon (VISA‐P, limb specific) [32] and International Knee Documentation Committee (IKDC) [33] surveys, and performed knee extensor strength testing on an electromechanical dynamometer (Biodex System 4, Biodex Medical Systems) at 60° of knee flexion for reporting of participant demographics [34].

### 2.2 Magnetic Resonance Imaging

**Figure 1:**
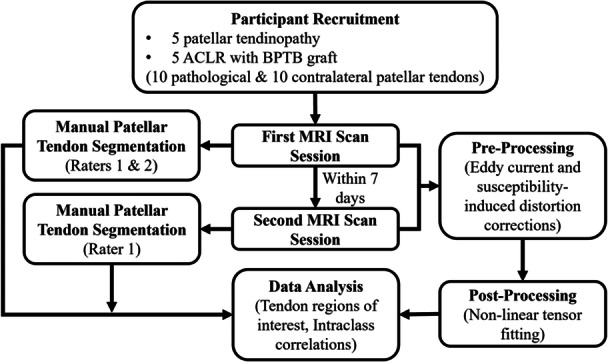
Study methodology to assess diffusion tensor imaging repeatability and reliability in pathological and contralateral patellar tendons in participants with patellar tendinopathy and anterior cruciate ligament reconstruction (ACLR) with bone‐patellar tendon‐bone (BPTB) grafts.

Two MRI scan sessions were collected within a 7‐day period separated by at least 24 h. Participants were asked to refrain from any strenuous exercise for the 24 h leading up to their imaging visits. With participants in a supine position and their legs extended, images were obtained bilaterally on a 3T SIGNA Premier (GE HealthCare, Chicago, IL, USA) utilizing a hard shell 18‐channel transmit/receive knee coil. 3D CUBE proton density weighted images (320 × 320 × 224 matrix, 0.5 × 0.5 ×0 .5 mm^3^ resolution, TR/TE = 1000/33.1 ms, ETL = 41) and DTI (axial oblique orientation, aligned with tendon, spin‐echo planar imaging (se‐EPI), 128 × 128 × 35 matrix, 1.4 × 1.4 × 3.0 mm^3^ resolution, TR/TE = 4008/60.8 ms, ASSET *R* = 2 acceleration, *b* = 800 s/mm^2^, 30 directions, four *B* = 0 encodings, AIR Recon DL processing = high, 2 signal averages, repeated twice with AP/PA reversed phase encode polarity, total averages = 4) were acquired for anatomical reference and tendon microstructure assessment (Figure 1 and Figure S1). The total DTI scan time was 9.5 min per limb. Pre‐processing including eddy current correction and susceptibility‐induced distortion correction were performed using EDDY [35] and FSL TOPUP [36] (FMRIB Software Library, OxCIN, Oxford, UK) followed by non‐linear tensor fitting with Camino Diffusion MRI Toolkit (UCL, London, UK) [37, 38, 39].

### 2.3 Patellar Tendon Segmentation

Using the 3D CUBE proton density weighted images obtained during the first MRI scan session, patellar tendons were manually segmented by both Rater 1 (EH) and Rater 2 (NI) (FSLeyes, v1.12.4, FSL, Analysis Group, FMRIB, Oxford, UK). The patellar tendon was segmented from the first slice of attachment at the patella to the attachment on the tibia [40]; if intratendinous calcifications were identified, they were segmented and excluded from the overall tendon mask. Rater 1 completed all segmentations using scans obtained during the second MRI scan session (Figure 1).

### 2.4 Tendon Regions of Interest

**Figure 2:**
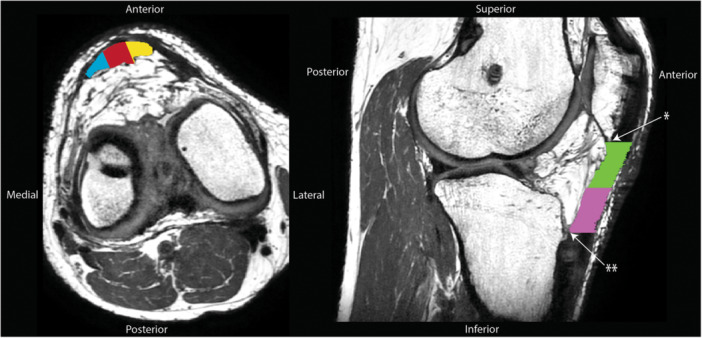
Proton density MRI overlaid with masks of the five patellar tendon boundaries for separating regions of interest. The free tendon was segmented from the distal end of the patella (*) to the attachment on the tibia (**). The tendon mask was then separated into five regions: (1) medial, (2) central, and (3) lateral regions by trisecting the tendon longitudinally into equal widths (left) and (4) proximal and (5) distal regions of equal length (right).

**Figure 3:**
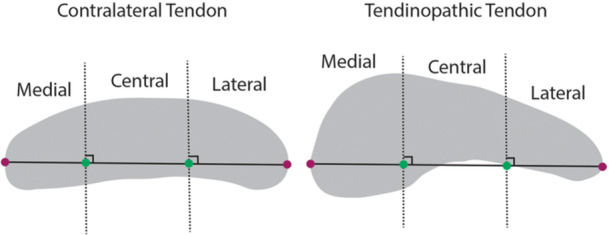
Transverse view of contralateral (left) and tendinopathic patellar tendons (right). To determine the medial, central, and lateral regions of interest, each tendon slice was trisected into equal widths along its longest cross‐sectional diameter. Perpendicular lines intersecting the trisection points determined the medial, central, and lateral region borders.

In addition to the whole tendon segmentation, five regions of interest were analyzed based on known regional differences in healthy patellar tendons [40], the high prevalence of tendinopathy observed in the proximal‐medial third of the tendon [17, 41], and common alterations in the central third from BPTB graft harvest [42]. Custom code (MATLAB, v2024b, Mathworks, Natick, MA) trisected the masked tendon longitudinally into full‐length *medial*, *central*, and *lateral* regions of equal width and separately split the tendon transversely into *proximal* and *distal* regions of equal lengths (Figure 2). For the longitudinal segmentations, the greatest cross‐sectional diameter of the tendon in transverse view was trisected into equal lengths. At the trisection points, perpendicular lines defined the boundaries of the medial, central, and lateral regions (Figure 3). This process was repeated for each tendon slice to create region‐specific tendon masks. Proximal and distal regions were determined by dividing the whole tendon mask length‐wise into two regions. If an odd number of slices existed, the middle slice was included in both the proximal and distal regions for analysis. Tendon volume was calculated by multiplying voxels identified within the tendon boundaries by voxel volume. Tendon masks were then down‐sampled to match the DTI resolution, eroded by one boundary pixel, and applied to their corresponding DTI scan.

### 2.5 DTI Scalar Metrics

DTI scalar metrics of interest included axial diffusivity (λ_1_), radial diffusivities (λ_2,_ λ_3_) perpendicular to λ_1_, and mean diffusivity (MD) to describe the magnitude of water molecule diffusion through tissue [28, 43], and fractional anisotropy (FA), a measure of the magnitude of diffusion directionality [44]. These parameters were calculated using FMRIB's Diffusion Toolbox (FMRIB Software Library, Oxford, UK) [45] for the whole tendon mask and within each of the five tendon regions of interest.

### 2.6 Statistical Analysis

Standard descriptive statistics for demographics including peak knee extensor torque, VISA‐P and IKDC scores were reported to characterize the study participants. Inter‐rater reliability of DTI variables of interest (λ_1_, λ_2_, λ_3_, MD, FA) extracted from tendon regions of interest and tendon volumes were calculated using intraclass correlations (ICCs) derived from linear mixed effects models for each variable by region and by limb (pathological and contralateral tendons). For participants with bilateral patellar tendinopathy, the more symptomatic tendon, as reported by the participant, was considered the pathological limb. The rater (Rater 1 vs. Rater 2) was modeled as a fixed effect and participants were modeled as random effects. ICCs for test‐retest repeatability were calculated similarly for each variable by region and by limb, with session (first scan vs. second scan) modeled as a fixed effect and participants modeled as random effects. To begin exploring the overall ICCs regardless of limb type (i.e., healthy, tendinopathy, BPTB), ICCs using a model that accounted for the fixed effects of limb type were also calculated. Confidence intervals (CIs) for ICCs were calculated using a bootstrapping method (*n* = 1000). ICC values less than 0.50 were considered poor agreement, between 0.50 and 0.75 moderate, between 0.75 and 0.90 good, and greater than 0.90 were excellent agreement [46]. To establish good reliability (ICC ≥ 0.75) between 2 ratings with 80% power and *α* set to 0.05, a sample size of 10 was calculated. To assess the variation in measures, standard error of measurements (SEMs, $\mathrm{SEM}=\mathrm{standard}\;\mathrm{deviation}*\sqrt{1-\mathrm{ICC}}$) were also calculated for each DTI metric by region within both pathological and contralateral tendons.

## 3 Results

Five participants (2 female, 3 males) with symptomatic patellar tendinopathy (age [mean(SD)] = 25.6 (6.2) years, mass = 74.5 (14.3) kg, height = 1.71 (0.12) m, symptom duration = 97.5 (72.4) months) and five (3 female, 2 males) after ACLR with BPTB autograft harvest (age = 22.8 (1.7) years, mass = 71.2 (11.9) kg, height = 1.69 (0.12) m, 24.6 (19.9) months since surgery) were enrolled in this study (Table 1). The second MRI scan session occurred within 3.4 (1.8) days of the first MRI scan session.

**Table 1:** Peak knee extensor torque, Victorian Institute of Sports Assessment—Patellar Tendon (VISA‐P, limb specific), and International Knee Documentation Committee (IKDC) scores [mean (SD)] separated by participants with patellar tendinopathy and anterior cruciate ligament reconstruction (ACLR) with bone‐patellar tendon‐bone (BPTB) autograft harvest.

Pathology	Tendon	Peak torque (Nm)	VISA‐P	IKDC score
Tendinopathy	Pathological	150.4 (24.6)	68.2 (16.4)	76.3 (12.1)
	Contralateral	181.2 (43.2)	71.6 (12.8)	
BPTB	Pathological	201.6 (29.9)	75.1 (6.5)	82.8 (7.3)
	Contralateral	258.5 (71.3)	91.6 (8.1)	

### 3.1 Inter‐Rater Reliability

ICCs of DTI scalar metrics from the first MRI scan session between raters ranged from 0.935 to 0.981 for λ_1_, 0.920–0.980 for λ_2_, 0.921–0.982 for λ_3_, 0.924–0.980 for MD, and 0.964–0.994 for FA across pathological patellar tendon regions. For contralateral patellar tendons, ICCs ranged from 0.928 to 0.967 for λ_1_, 0.935–0.965 for λ_2_, 0.935–0.963 for λ_3_, 0.932–0.966 for MD and 0.988–0.994 for FA across tendon regions. Tendon mask volume ICCs ranged from 0.859 to 0.956 for pathological and 0.856–0.935 for contralateral tendons (Table 2). DTI metric SEMs ranged from 0.020 × 10^−3^ mm^2^/s to 0.056 × 10^−3^ mm^2^/s for diffusivities and 0.006 to 0.019 for FA across regions in pathological tendons (Table S1). For contralateral tendons, SEMs ranged from 0.029 × 10^−3^ mm^2^/s to 0.060 × 10^−3^ mm^2^/s for diffusivities and from 0.008 to 0.013 for FA across regions.

**Table 2:** Inter‐rater reliability results using intraclass correlation (ICC) measures and confidence intervals (CIs) [ICC(CI)] for diffusion tensor imaging (DTI) diffusivities (λ_1_, λ_2,_ and λ_3_), mean diffusivity (MD), fractional anisotropy (FA), and mask volume across regions within pathological and contralateral patellar tendons.

Region	DTI metric	Pathological	Contralateral
Whole tendon	λ_1_	0.962 (0.956, 1.000)	0.947 (0.940, 0.999)
	λ_2_	0.953 (0.950, 1.000)	0.949 (0.945, 1.000)
	λ_3_	0.951 (0.942, 1.000)	0.951 (0.948, 1.000)
	MD	0.955 (0.951, 1.000)	0.948 (0.943, 1.000)
	FA	0.984 (0.969, 1.000)	0.993 (0.993, 1.000)
	Mask volume	0.927 (0.878, 0.999)	0.897 (0.900, 0.996)
Medial	λ_1_	0.952 (0.951, 1.000)	0.963 (0.953, 0.999)
	λ_2_	0.939 (0.934, 1.000)	0.962 (0.955, 1.000)
	λ_3_	0.932 (0.926, 1.000)	0.963 (0.953, 0.999)
	MD	0.941 (0.937, 1.000)	0.963 (0.953, 0.999)
	FA	0.964 (0.965, 1.000)	0.988 (0.988, 1.000)
	Mask volume	0.859 (0.833, 0.997)	0.886 (0.887, 0.997)
Central	λ_1_	0.935 (0.922, 1.000)	0.947 (0.944, 0.999)
	λ_2_	0.920 (0.919, 1.000)	0.950 (0.948, 0.999)
	λ_3_	0.921 (0.915, 0.999)	0.946 (0.943, 0.999)
	MD	0.924 (0.922, 1.000)	0.947 (0.944, 0.999)
	FA	0.977 (0.965, 1.000)	0.992 (0.993, 1.000)
	Mask volume	0.924 (0.889, 0.998)	0.900 (0.905, 0.996)
Lateral	λ_1_	0.979 (0.970, 0.999)	0.947 (0.954, 1.000)
	λ_2_	0.973 (0.965, 0.999)	0.946 (0.956, 1.000)
	λ_3_	0.976 (0.968, 1.000)	0.955 (0.963, 1.000)
	MD	0.976 (0.969, 0.999)	0.947 (0.957, 1.000)
	FA	0.994 (0.975, 1.000)	0.994 (0.994, 1.000)
	Mask volume	0.956 (0.914, 0.999)	0.912 (0.908, 0.998)
Proximal	λ_1_	0.953 (0.931, 0.999)	0.928 (0.922, 1.000)
	λ_2_	0.940 (0.934, 0.999)	0.935 (0.932, 1.000)
	λ_3_	0.937 (0.927, 1.000)	0.935 (0.930, 1.000)
	MD	0.944 (0.933, 0.999)	0.932 (0.928, 1.000)
	FA	0.974 (0.935, 1.000)	0.988 (0.986, 1.000)
	Mask volume	0.898 (0.883, 0.997)	0.856 (0.854, 0.997)
Distal	λ_1_	0.981 (0.976, 1.000)	0.967 (0.968, 1.000)
	λ_2_	0.980 (0.977, 1.000)	0.965 (0.967, 1.000)
	λ_3_	0.982 (0.978, 1.000)	0.968 (0.970, 1.000)
	MD	0.980 (0.976, 1.000)	0.966 (0.967, 1.000)
	FA	0.991 (0.989, 1.000)	0.994 (0.995, 1.000)
	Mask volume	0.944 (0.871, 0.999)	0.935 (0.933, 0.998)

### 3.2 Test‐Retest Repeatability

ICCs of DTI scalar metrics comparing first and second MRI scan sessions ranged from 0.164 to 0.477 for λ_1_, 0.222–0.409 for λ_2_, 0.230–0.411 for λ_3_, 0.212–0.388 for MD, and 0.411–0.709 for FA across pathological patellar tendon regions. For the contralateral patellar tendon regions, ICC values ranged from 0.689 to 0.824 for λ_1_, 0.635–0.829 for λ_2_, 0.570–0.842 for λ_3_, 0.630–0.830 for MD, and 0.566–0.842 for FA. Tendon mask volume ICCs ranged from 0.933 to 0.979 and 0.962–0.992 for pathological and contralateral tendon regions respectively. Since one major outlier was identified in the test‐retest repeatability analysis (Figure S2), pathological tendon ICCs were also re‐calculated after removal (n = 9). Recalculated pathological ICCs ranged from 0.471 to 0.755 across all variables of interest (Table 3). For pathological tendons, DTI metric SEM values ranged from 0.143 × 10^−3^ mm^2^/s to 0.221 × 10^−3^ mm^2^/s for diffusivities and from 0.051 to 0.063 for FA across tendon regions. SEM values ranged from 0.070 × 10^−3^ mm^2^/s to 0.129 × 10^−3^ mm^2^/s for diffusivities and from 0.041 to 0.059 for FA across all contralateral tendon regions. With the outlier removed, pathological SEM values ranged from 0.074 × 10^−3^ mm^2^/s to 0.151 × 10^−3^ mm^2^/s for diffusivities and from 0.038 to 0.049 for FA (Table S2). The pooled ICCs with the outlier removed ranged from 0.469 to 0.809 for λ_1_, 0.584–0.876 for λ_2_, 0.513–0.903 for λ_3_, 0.545–0.871 for MD, and 0.577–0.905 for FA across tendon regions (Table S3).

**Table 3:** Test‐retest repeatability results using intraclass correlation (ICC) measures and confidence intervals (CIs) [ICC(CI)] for diffusion tensor imaging (DTI) diffusivities (λ_1_, λ_2,_ and λ_3_), mean diffusivity (MD), fractional anisotropy (FA), and mask volume across regions within pathological (*N* = 10) and contralateral (*N* = 10) patellar tendons. Exclusion of an outlier pathological tendon improved ICCs for the pathological patellar tendons (*N* = 9).

Region	DTI metric	Pathological (*N* = 10)	Pathological (*N* = 9)	Contralateral (*N* = 10)
Whole tendon	λ_1_	0.252 (0.164, 0.994)	0.608 (0.611, 0.998)	0.798 (0.762, 0.996)
	λ_2_	0.253 (0.091, 0.996)	0.597 (0.579, 0.998)	0.810 (0.803, 0.997)
	λ_3_	0.230 (0.000, 0.994)	0.562 (0.498, 0.996)	0.821 (0.820, 0.997)
	MD	0.234 (0.103, 0.996)	0.585 (0.570, 0.998)	0.808 (0.797, 0.998)
	FA	0.527 (0.346, 0.988)	0.722 (0.725, 1.000)	0.812 (0.812, 0.999)
	Mask volume	0.971 (0.958, 1.000)	0.969 (0.952, 1.000)	0.983 (0.981, 1.000)
Medial	λ_1_	0.272 (0.204, 0.993)	0.489 (0.312, 0.993)	0.814 (0.801, 0.998)
	λ_2_	0.374 (0.221, 0.995)	0.590 (0.439, 0.996)	0.829 (0.827, 0.998)
	λ_3_	0.411 (0.210, 0.998)	0.648 (0.515, 1.000)	0.842 (0.844, 0.998)
	MD	0.338 (0.206, 0.997)	0.576 (0.422, 0.997)	0.830 (0.829, 0.998)
	FA	0.709 (0.639, 0.996)	0.874 (0.867, 0.998)	0.842 (0.851, 0.998)
	Mask volume	0.933 (0.913, 1.000)	0.926 (0.903, 1.000)	0.971 (0.970, 0.999)
Central	λ_1_	0.220 (0.112, 0.991)	0.600 (0.610, 0.995)	0.802 (0.791, 0.999)
	λ_2_	0.222 (0.098, 0.991)	0.614 (0.627, 0.998)	0.823 (0.825, 0.998)
	λ_3_	0.238 (0.066, 0.995)	0.599 (0.603, 0.996)	0.834 (0.844, 0.998)
	MD	0.212 (0.092, 0.991)	0.598 (0.611, 0.994)	0.819 (0.828, 0.998)
	FA	0.411 (0.149, 0.986)	0.604 (0.594, 0.997)	0.823 (0.811, 0.999)
	Mask volume	0.979 (0.975, 1.000)	0.980 (0.975, 1.000)	0.962 (0.958, 1.000)
Lateral	λ_1_	0.477 (0.346, 0.997)	0.738 (0.742, 0.998)	0.689 (0.612, 0.997)
	λ_2_	0.409 (0.234, 0.995)	0.669 (0.656, 0.998)	0.635 (0.512, 0.997)
	λ_3_	0.244 (0.000, 0.995)	0.502 (0.400, 0.998)	0.570 (0.454, 0.995)
	MD	0.388 (0.210, 0.996)	0.654 (0.635, 0.997)	0.630 (0.517, 0.998)
	FA	0.514 (0.279, 0.990)	0.616 (0.536, 0.999)	0.566 (0.488, 0.989)
	Mask volume	0.970 (0.949, 1.000)	0.967 (0.943, 0.999)	0.992 (0.993, 1.000)
Proximal	λ_1_	0.403 (0.322, 0.989)	0.630 (0.637, 0.996)	0.783 (0.739, 0.996)
	λ_2_	0.337 (0.219, 0.988)	0.567 (0.556, 0.996)	0.808 (0.780, 0.998)
	λ_3_	0.285 (0.096, 0.992)	0.471 (0.371, 0.998)	0.833 (0.813, 0.997)
	MD	0.343 (0.247, 0.990)	0.561 (0.555, 0.996)	0.808 (0.779, 0.997)
	FA	0.542 (0.023, 0.996)	0.644 (0.320, 0.998)	0.816 (0.806, 0.996)
	Mask volume	0.970 (0.965, 1.000)	0.968 (0.962, 1.000)	0.980 (0.980, 1.000)
Distal	λ_1_	0.164 (0.000, 0.993)	0.687 (0.629, 0.996)	0.824 (0.798, 0.996)
	λ_2_	0.292 (0.000, 0.995)	0.739 (0.702, 0.995)	0.813 (0.796, 0.996)
	λ_3_	0.280 (0.069, 0.995)	0.755 (0.726, 0.998)	0.797 (0.799, 0.998)
	MD	0.220 (0.000, 0.994)	0.724 (0.678, 0.995)	0.813 (0.797, 0.995)
	FA	0.447 (0.361, 0.997)	0.688 (0.679, 0.998)	0.717 (0.703, 0.997)
	Mask volume	0.966 (0.937, 1.000)	0.964 (0.933, 1.000)	0.985 (0.982, 1.00)

## 4 Discussion

The purpose of this study was to determine the reliability of DTI scalar metrics obtained from patellar tendon segmentations from independent raters and the repeatability of measuring DTI scalar metrics in both pathological and contralateral patellar tendons on separate days. Excellent inter‐rater reliability was observed for all DTI metrics within all tendon regions of interest. DTI metric repeatability was moderate to good in contralateral patellar tendons, but poor to moderate in pathological tendons. When a pathological tendon outlier was removed, however, moderate repeatability was generally seen across DTI metrics and regions in pathological tendons. Pathological and contralateral tendon mask volumes demonstrated good to excellent agreement across raters and between scan sessions as expected. DTI may offer a reliable non‐invasive assessment of patellar tendon microstructure to provide clinicians and researchers insight into patellar tendon pathophysiology.

### 4.1 Sensitivity of DTI Scalar Metrics to Minor Differences in Segmentations

To date, no inter‐rater reliability of DTI metrics have been reported for tendons, and particularly for tendons with pathology. DTI scalar metrics from our inter‐rater reliability analysis demonstrated better reliability than the tendon mask volume reliability. A similar DTI reliability study applying manual segmentation to healthy hamstring muscles reported excellent DTI metric similarity between raters [47]. Across two independent raters, DTI scalar metrics were found to have excellent agreement (ICC values ranged from 0.920 to 0.994) for both pathological and contralateral patellar tendons across tendon regions, demonstrating low sensitivity to rater‐specific variability in segmentations. Within each tendon region, FA consistently had the greatest ICC for both pathological and contralateral tendons. Good to excellent inter‐rater reliability (average ICC values ranged from 0.856 to 0.956) between tendon region volumes was also observed. These ICCs and the low SEM values (0.020–0.060 × 10^−3^ mm^2^/s for diffusivities and 0.006 to 0.019 for FA) confirm the reliability of our method across multiple raters and that it is not sensitive to subtle differences in segmentations.

### 4.2 Test‐Retest Repeatability of Patellar Tendon DTI

DTI scalar metric repeatability across scan sessions was found to be moderate to good (ICC values ranging from 0.566 to 0.842 with SEM values ranging from 0.070 × 10^−3^ mm^2^/s to 0.129 × 10^−3^ mm^2^/s for diffusivities and from 0.041 to 0.059 for FA) in the contralateral patellar tendons. These ICC ranges are on the same order of magnitude as those reported in a previous study assessing hamstring muscle test‐retest repeatability in DTI metrics [47]. Interestingly, the lateral patellar tendon region produced only moderate DTI metric agreement (0.566–0.689) between time points whereas all other regions produced moderate to good agreement (0.717–0.842) in contralateral tendons. Such findings may be due to regional differences known in the patellar tendon structure [34, 40, 42], however, further analysis is warranted.

For pathological patellar tendons, repeatability was found to be poor to moderate with ICC values ranging from 0.164 to 0.709 (SEM values from 0.143 to 0.221 × 10^−3^ mm^2^/s for diffusivities and 0.051–0.063 for FA) across the reported scalar DTI metrics and tendon regions. FA measures had better agreement between the first and second scans than the diffusivity measures within pathological tendons. Even with consistent tendon volumes across scan sessions, one pathological tendon presented with exceptionally different DTI metrics between the first and second MRI scan sessions (Figure S2), hence, additional analysis with this one outlier removed is also reported. Due to the small sample, this one outlier had a major impact on the ICC values. DTI metric discrepancies between the pathological and contralateral tendons across scan sessions may be due to potentially greater daily microstructural fluctuations in pathological patellar tendons. Possible biological mechanisms for these discrepancies may be attributed to greater, and more frequent collagen turnover known to occur in tendons with tendinopathy, or greater fluctuations in water content due to higher volumes of extracellular matrix, potentially resulting in greater interstitial fluid flow [16]. Given the small volume and relatively short T2 relaxation time of the patellar tendon, artifacts in MRI acquisition such as motion, incomplete fat suppression, or susceptibility artifacts may also be contributing to the observed discrepancies. Further investigation is needed to understand the origin of the pathological and contralateral tendon discrepancies.

### 4.3 Trends in Pathological Patellar Tendon Scalar DTI Metrics

**Figure 4:**
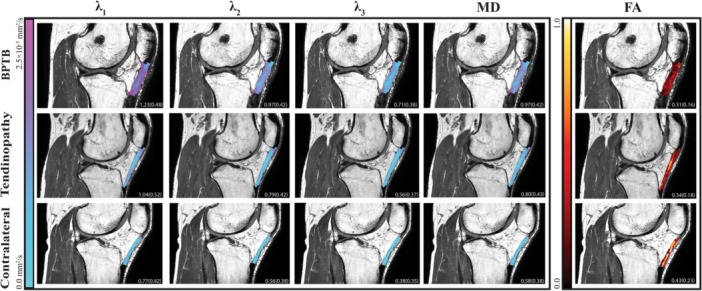
Diffusion scalar maps of diffusivity (λ_1_, λ_2_, λ_3_), mean diffusivity (MD) and fractional anisotropy (FA) measures overlaid with proton density MRI of a patellar tendon 8‐months after bone‐patellar tendon‐bone (BPTB) autograft harvest, a tendon with tendinopathy, and a contralateral patellar tendon. Mean and standard deviation of the scalar metric included in the bottom right corner of each image.

In contrast to the contralateral tendons, the pathological tendons had higher diffusivity and lower FA (poorer tissue organization), possibly reflecting loosely packed collagen fibers and greater extracellular matrix spacing commonly observed in healing tendons (Figure 4, Table S4). Patellar tendons after BPTB graft presented with greater diffusivity measures and tendon volumes across regions, in agreement with the macrostructural healing process observed [11, 18, 20, 42]. Results from our small sample also indicate greater average diffusivities within the medial and proximal regions of tendinopathic patellar tendon, the regions where tendinopathy is most commonly observed at the macrostructural level [17, 41].

Compared to previously reported healthy patellar tendon scalar metrics obtained using similar methods, our results in the contralateral tendon measured at lower axial (0.922 vs. 1.54 10^−3^mm^2^/s) and mean (0.693 vs. 1.18 10^−3^ mm^2^/s) diffusivities and slightly higher FA (0.41 vs. 0.30) [31]. Observations of the raw image data suggest that signal‐to‐noise ratio bias was not driving these results. These discrepancies may be due to differences in knee positioning (fully extended knee vs. a flexed knee to position the patellar tendon at 55° with respect to the magnet) during imaging. Studies using shear wave elastography, an ultrasound‐based method for quantifying tendon stiffness, have observed knee angle dependent measurements in the patellar tendon, likely due to the difference in passive tension altering extracellular matrix and microstructural spacing through the tendon [48, 49]. The previous study also had an older average participant age and only restricted exercise up to 2 h prior to imaging vs. 24 h. Further investigation into the influence of knee joint position, activity, and scan parameters on DTI metrics may be warranted. Our method is repeatable and seems to capture region and pathology specific characteristics that are known about tendon, hence, we are confident in the application of our methods in the future.

### 4.4 Limitations

Our limited sample must be acknowledged, leading to the large CIs observed in our ICCs, particularly in the pathological tendons where point estimates were lower. Bootstrapping methods are also known to establish conservative CIs, particularly with smaller sample sizes, and the ranges should be interpreted with caution. Slight variations in participant positioning in the scanner and the resulting alignment of tendon fibers relative to the main magnetic field of the scanner may have influenced the repeatability measures. Findings from the contralateral tendons, however, give us very strong confidence that the methodological repeatability is high, and that findings in the pathological tendons may be an artifact of the pathology itself rather than limitations to our methods. Despite the small sample size, these preliminary findings are encouraging for future implementation of DTI for tendon research. Four of the five participants with tendinopathy presented with bilateral symptoms, and a contralateral limb from participants with strictly unilateral symptoms may have also resulted in even higher ICCs. Tissue‐level characteristics, such as increased water content in contralateral tendons with tendinopathy, may affect MRI contrasts, resulting in differences in DTI scalar metrics between scan sessions, thus influencing the repeatability measures. Additionally, the tendinopathic tendon for one participant's second scan produced DTI metrics vastly different from the first MRI scan. As the investigation by an expert medical physicist (SAH) found no issues with the data collection or analysis processing, the data are included for completeness of reporting our results.

## 5 Conclusion

Excellent inter‐rater reliability was observed for all DTI metrics within pathological and contralateral patellar tendons when segmentations were performed by different raters on the same scans. Repeatability was also moderate to good in healthy patellar tendons, however, it was worse for pathological tendons. The difference in test‐retest findings between the pathological and contralateral tendons we observed may reflect the difference in between‐day fluctuations of the DTI scalar metrics within the tendons, rather than poor repeatability of the methods. While clinical implications of altered DTI scalar metrics in pathological tendons require further investigation, findings from this study provide clinicians and researchers with a reliable method for capturing patellar tendon microstructure.

## Author Contributions

E.A.S. contributed to the analysis, interpretation, drafting of the manuscript, and critical review. E.H. assisted with data processing and critical review. D.O. contributed to the data acquisition and critical review. D.H., K.S.L., and B.C.H. contributed to the funding acquisition, research design, interpretation, supervision, and critical review. S.A.H. and N.I. contributed to the funding acquisition, research design, data interpretation, drafting of the manuscript, and critical review. All authors have read and approved the final version prior to submission.

## Disclosure

The University of Wisconsin Radiology MRI Research Group (Hernando, Hurley) receives support from GE HealthCare. Dr. Bryan Heiderscheit has received research support (to institution) and is an advisor to GE HealthCare. Dr. Kenneth Lee has received research support from GE HealthCare.

## Supporting information


**Figure S1:** Example images showing single axial b=0 (top‐left), average b = 0 (averaged over 4 B0 images) (top‐middle) and b=800 (averaged over 30 directions) (top‐right) showing raw diffusion signal, along with representative signal from a signal voxel (green crosshair) showing signal level vs. diffusion encoding (bottom).

**Figure S2:** Scatter plots comparing first and second scan measurements of diffusion tensor imaging (DTI) diffusivities (λ_1_, λ_2_, and λ_3_), mean diffusivity (MD), fractional anisotropy (FA), and mask volume for whole pathological and contralateral patellar tendon masks.

**Table S1:** Intraclass correlations (ICCs), pooled mean and standard deviations (SDs), and calculated standard error of measurements (SEMs) for diffusion tensor imaging (DTI) diffusivities (λ_1_, λ_2_, and λ_3_) [10^−3^mm^2^/s], mean diffusivity (MD) [10^−3^mm^2^/s], fractional anisotropy (FA) [values range from 0 to 1], and mask volume [cm^3^] across raters for pathological and contralateral tendon regions.

**Table S2:** Intraclass correlations (ICCs), pooled mean and standard deviations (SDs), and calculated standard error of measurements (SEMs) for diffusion tensor imaging (DTI) diffusivities (λ_1_, λ_2_, and λ_3_) [10^−3^mm^2^/s], mean diffusivity (MD) [10^−3^mm^2^/s], fractional anisotropy (FA) [values range from 0 to 1], and mask volume [cm^3^] between the first and second MRI scan sessions for pathological and contralateral tendon regions. ICCs, means, SDs, and SEMs were recalculated for the pathological tendon regions with the outlier removed (N = 9).

**Table S3:** Test‐retest repeatability intraclass correlation (ICC) results pooling all limbs and including limb type (healthy, tendinopathy, BPTB) as a fixed effect to model overall ICC measures and calculate confidence intervals (CIs) [ICC(CI)] for diffusion tensor imaging (DTI) diffusivities (λ_1_, λ_2_, and λ_3_), mean diffusivity (MD), fractional anisotropy (FA), and mask volume across tendon regions.

**Table S4:** Mean and standard deviation (SD) measures of diffusion tensor imaging (DTI) diffusivities (λ_1_, λ_2_, and λ_3_) [10^−3^mm^2^/s], mean diffusivity (MD) [10^−3^mm^2^/s], fractional anisotropy (FA), and mask volume [cm^3^] across regions within pathological and contralateral patellar tendons segmented by Rater 1 from the first scan session.
