# Supplementary material for: Reliability and Repeatability of Diffusion Tensor Imaging in Healthy and Pathological Patellar Tendons
Source: J Orthop Res. 2026 Jan 29;44(2):e70156. doi: 10.1002/jor.70156 (PMC12853323; doi:10.1002/jor.70156)

**Figure S-1.** Example images showing single axial b=0 (top-left), average b = 0 (averaged over 4 B0 images) (top-middle) and b=800 (averaged over 30 directions) (top-right) showing raw diffusion signal, along with representative signal from a signal voxel (green crosshair) showing signal level vs. diffusion encoding (bottom).
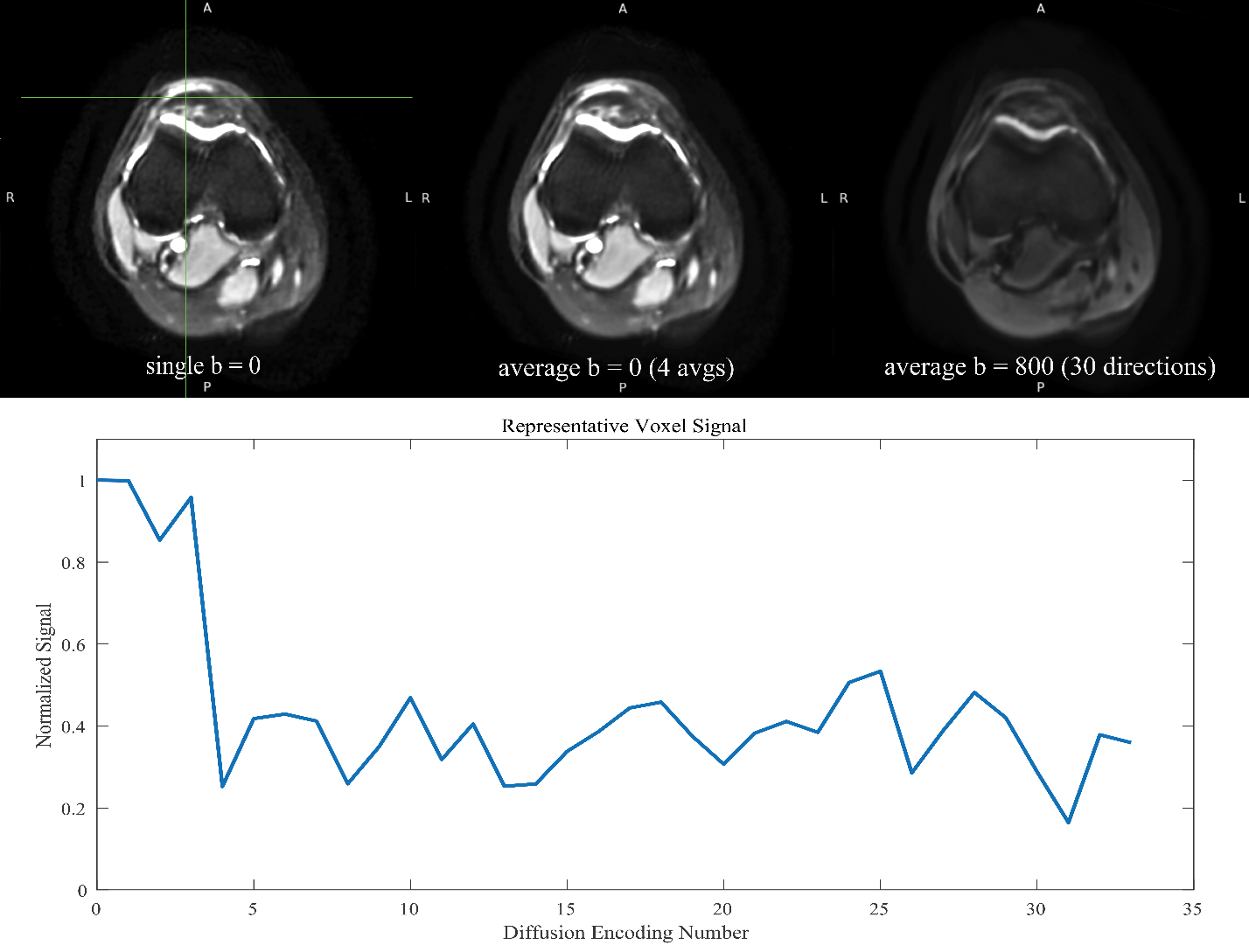

Supplement: Supplementary file 1 — Figure S1: Example images showing single axial b=0 (top‐left), average b = 0 (averaged over 4 B0 images) (top‐middle) and b=800 (averaged over 30 directions) (top‐right) showing raw diffusion signal, along with representative signal from a signal voxel (green crosshair) showing signal level vs. diffusion encoding (bottom). [file JOR-44-0-s005.docx]
