# Supplementary material for: Reliability and Repeatability of Diffusion Tensor Imaging in Healthy and Pathological Patellar Tendons
Source: J Orthop Res. 2026 Jan 29;44(2):e70156. doi: 10.1002/jor.70156 (PMC12853323; doi:10.1002/jor.70156)

**Figure S-2.** Scatter plots comparing first and second scan measurements of diffusion tensor imaging (DTI) diffusivities (λ_1_, λ_2,_ and λ_3_), mean diffusivity (MD), fractional anisotropy (FA), and mask volume for whole pathological and contralateral patellar tendon masks.


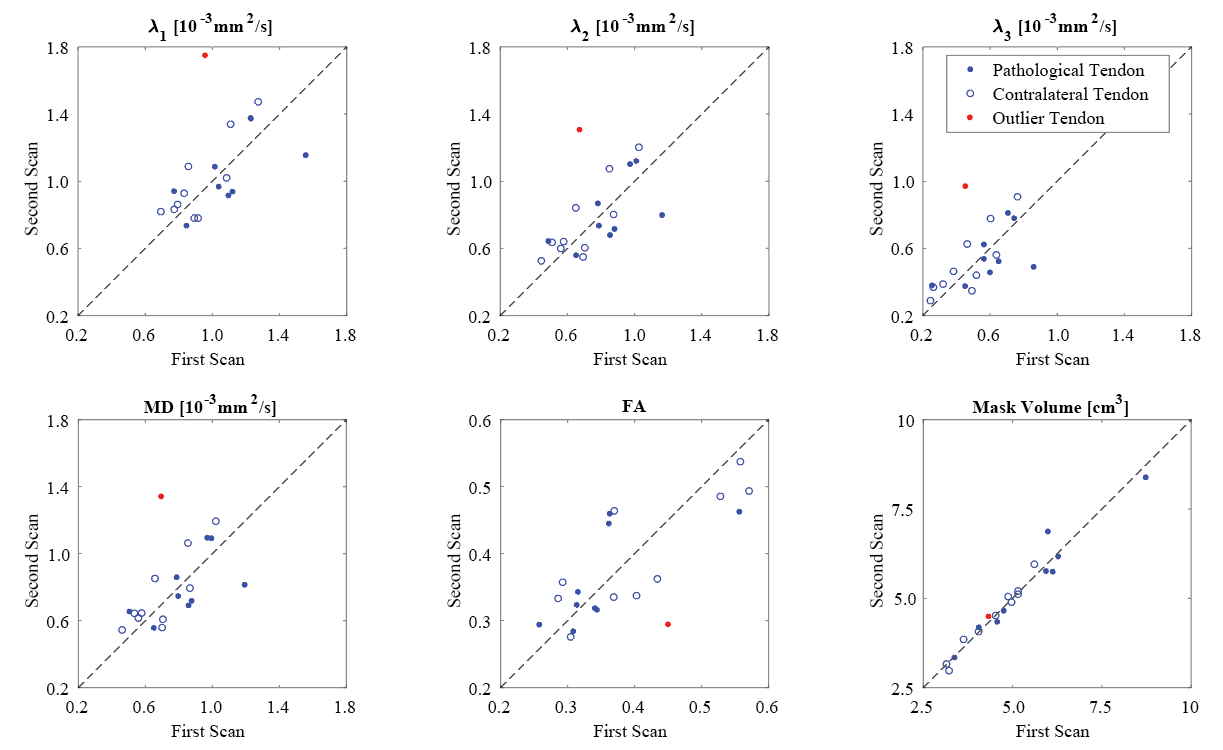

Supplement: Supplementary file 2 — Figure S2: Scatter plots comparing first and second scan measurements of diffusion tensor imaging (DTI) diffusivities (λ1, λ2, and λ3), mean diffusivity (MD), fractional anisotropy (FA), and mask volume for whole pathological and contralateral patellar tendon masks. [file JOR-44-0-s006.docx]
