# Supplementary material for: Reliability and Repeatability of Diffusion Tensor Imaging in Healthy and Pathological Patellar Tendons
Source: J Orthop Res. 2026 Jan 29;44(2):e70156. doi: 10.1002/jor.70156 (PMC12853323; doi:10.1002/jor.70156)
Supplement: Supplementary file 3 — Table S1: Intraclass correlations (ICCs), pooled mean and standard deviations (SDs), and calculated standard error of measurements (SEMs) for diffusion tensor imaging (DTI) diffusivities (λ1, λ2, and λ3) [10−3mm2/s], mean diffusivity (MD) [10−3mm2/s], fractional anisotropy (FA) [values range from 0 to 1], and mask volume [cm3] across raters for pathological and contralateral tendon regions. [file JOR-44-0-s003.docx]

**Table S-1.** Intraclass correlations (ICCs), pooled mean and standard deviations (SDs), and calculated standard error of measurements (SEMs) for diffusion tensor imaging (DTI) diffusivities (λ_1_, λ_2,_ and λ_3_) [10^-3^mm^2^/s], mean diffusivity (MD) [10^-3^mm^2^/s], fractional anisotropy (FA) [values range from 0 to 1], and mask volume [cm^3^] across raters for pathological and contralateral tendon regions.

|  |  | **Pathological** | | | **Contralateral** | | |
| --- | --- | --- | --- | --- | --- | --- | --- |
| **Tendon Region** | **DTI Metric** | **ICC** | **Mean(SD)** | **SEM** | **ICC** | **Mean(SD)** | **SEM** |
| Whole Tendon | λ_1_ | 0.962 | 1.110(0.205) | 0.040 | 0.947 | 0.951(0.194) | 0.045 |
|  | λ_2_ | 0.953 | 0.850(0.182) | 0.039 | 0.949 | 0.717(0.197) | 0.044 |
|  | λ_3_ | 0.951 | 0.604(0.161) | 0.036 | 0.951 | 0.494(0.183) | 0.041 |
|  | MD | 0.955 | 0.855(0.181) | 0.038 | 0.948 | 0.721(0.190) | 0.043 |
|  | FA | 0.984 | 0.356(0.080) | 0.010 | 0.993 | 0.406(0.106) | 0.009 |
|  | Mask Volume | 0.927 | 5.611(1.504) | 0.407 | 0.897 | 4.626(0.851) | 0.273 |
| Medial | λ_1_ | 0.952 | 1.104(0.185) | 0.041 | 0.963 | 1.037(0.248) | 0.047 |
|  | λ_2_ | 0.939 | 0.842(0.187) | 0.046 | 0.962 | 0.801(0.254) | 0.049 |
|  | λ_3_ | 0.932 | 0.605(0.187) | 0.049 | 0.963 | 0.553(0.243) | 0.047 |
|  | MD | 0.941 | 0.850(0.185) | 0.045 | 0.963 | 0.797(0.247) | 0.048 |
|  | FA | 0.964 | 0.377(0.099) | 0.019 | 0.988 | 0.407(0.110) | 0.012 |
|  | Mask Volume | 0.859 | 1.258(0.353) | 0.133 | 0.886 | 1.140(0.196) | 0.066 |
| Central | λ_1_ | 0.935 | 1.073(0.192) | 0.049 | 0.947 | 0.904(0.207) | 0.048 |
|  | λ_2_ | 0.920 | 0.816(0.171) | 0.048 | 0.950 | 0.671(0.208) | 0.046 |
|  | λ_3_ | 0.921 | 0.580(0.152) | 0.043 | 0.946 | 0.457(0.188) | 0.044 |
|  | MD | 0.924 | 0.823(0.169) | 0.047 | 0.947 | 0.677(0.200) | 0.046 |
|  | FA | 0.977 | 0.360(0.079) | 0.012 | 0.992 | 0.425(0.113) | 0.010 |
|  | Mask Volume | 0.924 | 2.344(0.682) | 0.188 | 0.900 | 1.823(0.351) | 0.111 |
| Lateral | λ_1_ | 0.979 | 1.160(0.285) | 0.041 | 0.947 | 0.964(0.194) | 0.045 |
|  | λ_2_ | 0.973 | 0.899(0.242) | 0.040 | 0.946 | 0.730(0.189) | 0.044 |
|  | λ_3_ | 0.976 | 0.635(0.195) | 0.030 | 0.955 | 0.512(0.171) | 0.036 |
|  | MD | 0.976 | 0.898(0.239) | 0.037 | 0.947 | 0.735(0.181) | 0.042 |
|  | FA | 0.994 | 0.336(0.084) | 0.006 | 0.994 | 0.378(0.105) | 0.008 |
|  | Mask Volume | 0.956 | 2.009(0.512) | 0.107 | 0.912 | 1.666(0.341) | 0.101 |
| Proximal | λ_1_ | 0.953 | 1.182(0.254) | 0.055 | 0.928 | 0.983(0.223) | 0.060 |
|  | λ_2_ | 0.940 | 0.932(0.228) | 0.056 | 0.935 | 0.757(0.233) | 0.059 |
|  | λ_3_ | 0.937 | 0.685(0.207) | 0.052 | 0.935 | 0.537(0.216) | 0.055 |
|  | MD | 0.944 | 0.933(0.228) | 0.054 | 0.932 | 0.759(0.223) | 0.058 |
|  | FA | 0.974 | 0.324(0.082) | 0.013 | 0.988 | 0.387(0.118) | 0.013 |
|  | Mask Volume | 0.898 | 3.019(0.764) | 0.245 | 0.856 | 2.498(0.467) | 0.178 |
| Distal | λ_1_ | 0.981 | 1.022(0.177) | 0.025 | 0.967 | 0.920(0.183) | 0.033 |
|  | λ_2_ | 0.980 | 0.751(0.162) | 0.023 | 0.965 | 0.676(0.173) | 0.032 |
|  | λ_3_ | 0.982 | 0.507(0.146) | 0.020 | 0.968 | 0.449(0.158) | 0.029 |
|  | MD | 0.980 | 0.760(0.158) | 0.022 | 0.966 | 0.682(0.169) | 0.031 |
|  | FA | 0.991 | 0.394(0.091) | 0.009 | 0.994 | 0.428(0.100) | 0.008 |
|  | Mask Volume | 0.944 | 2.640(0.777) | 0.184 | 0.935 | 2.169(0.414) | 0.106 |
