# Supplementary material for: Reliability and Repeatability of Diffusion Tensor Imaging in Healthy and Pathological Patellar Tendons
Source: J Orthop Res. 2026 Jan 29;44(2):e70156. doi: 10.1002/jor.70156 (PMC12853323; doi:10.1002/jor.70156)
Supplement: Supplementary file 4 — Table S2: Intraclass correlations (ICCs), pooled mean and standard deviations (SDs), and calculated standard error of measurements (SEMs) for diffusion tensor imaging (DTI) diffusivities (λ1, λ2, and λ3) [10−3mm2/s], mean diffusivity (MD) [10−3mm2/s], fractional anisotropy (FA) [values range from 0 to 1], and mask volume [cm3] between the first and second MRI scan sessions for pathological and contralateral tendon regions. ICCs, means, SDs, and SEMs were recalculated for the pathological tendon regions with the outlier removed (N = 9). [file JOR-44-0-s001.docx]

**Table S-2.** Intraclass correlations (ICCs), pooled mean and standard deviations (SDs), and calculated standard error of measurements (SEMs) for diffusion tensor imaging (DTI) diffusivities (λ_1_, λ_2,_ and λ_3_) [10^-3^mm^2^/s], mean diffusivity (MD) [10^-3^mm^2^/s], fractional anisotropy (FA) [values range from 0 to 1], and mask volume [cm^3^] between the first and second MRI scan sessions for pathological and contralateral tendon regions. ICCs, means, SDs, and SEMs were recalculated for the pathological tendon regions with the outlier removed (N = 9).

|  |  | **Pathological (N = 10)** | | | **Pathological (N = 9)** | | | **Contralateral (N = 10)** | | |
| --- | --- | --- | --- | --- | --- | --- | --- | --- | --- | --- |
| **Tendon Region** | **DTI Metric** | **ICC** | **Mean(SD)** | **SEM** | **ICC** | **Mean(SD)** | **SEM** | **ICC** | **Mean(SD)** | **SEM** |
| Whole Tendon | λ_1_ | 0.252 | 1.104(0.251) | 0.217 | 0.608 | 1.070(0.208) | 0.130 | 0.798 | 0.957(0.205) | 0.092 |
|  | λ_2_ | 0.253 | 0.839(0.210) | 0.182 | 0.597 | 0.815(0.186) | 0.118 | 0.810 | 0.718(0.199) | 0.087 |
|  | λ_3_ | 0.230 | 0.590(0.176) | 0.155 | 0.562 | 0.570(0.157) | 0.104 | 0.821 | 0.493(0.178) | 0.075 |
|  | MD | 0.234 | 0.844(0.210) | 0.184 | 0.585 | 0.818(0.181) | 0.117 | 0.808 | 0.723(0.192) | 0.084 |
|  | FA | 0.527 | 0.358(0.075) | 0.051 | 0.722 | 0.361(0.075) | 0.040 | 0.812 | 0.405(0.094) | 0.041 |
|  | Mask Volume | 0.971 | 5.410(1.439) | 0.244 | 0.969 | 5.458(1.460) | 0.256 | 0.983 | 4.461(0.867) | 0.112 |
| Medial | λ_1_ | 0.272 | 1.077(0.258) | 0.221 | 0.489 | 1.038(0.200) | 0.143 | 0.814 | 1.050(0.298) | 0.129 |
|  | λ_2_ | 0.374 | 0.810(0.227) | 0.180 | 0.590 | 0.785(0.202) | 0.129 | 0.829 | 0.810(0.294) | 0.122 |
|  | λ_3_ | 0.411 | 0.572(0.208) | 0.160 | 0.648 | 0.551(0.192) | 0.114 | 0.842 | 0.559(0.280) | 0.111 |
|  | MD | 0.338 | 0.820(0.228) | 0.186 | 0.576 | 0.791(0.196) | 0.128 | 0.830 | 0.806(0.289) | 0.119 |
|  | FA | 0.709 | 0.382(0.105) | 0.057 | 0.874 | 0.386(0.107) | 0.038 | 0.842 | 0.409(0.102) | 0.041 |
|  | Mask Volume | 0.933 | 1.206(0.329) | 0.085 | 0.926 | 1.212(0.336) | 0.092 | 0.971 | 1.090(0.203) | 0.034 |
| Central | λ_1_ | 0.220 | 1.073(0.245) | 0.217 | 0.600 | 1.038(0.199) | 0.126 | 0.802 | 0.904(0.204) | 0.091 |
|  | λ_2_ | 0.222 | 0.812(0.202) | 0.178 | 0.614 | 0.787(0.175) | 0.109 | 0.823 | 0.662(0.198) | 0.083 |
|  | λ_3_ | 0.238 | 0.571(0.168) | 0.147 | 0.599 | 0.551(0.148) | 0.094 | 0.834 | 0.445(0.172) | 0.070 |
|  | MD | 0.212 | 0.818(0.203) | 0.180 | 0.598 | 0.792(0.172) | 0.109 | 0.819 | 0.670(0.189) | 0.081 |
|  | FA | 0.411 | 0.358(0.067) | 0.052 | 0.604 | 0.361(0.068) | 0.043 | 0.823 | 0.430(0.103) | 0.043 |
|  | Mask Volume | 0.979 | 2.281(0.667) | 0.097 | 0.980 | 2.303(0.677) | 0.096 | 0.962 | 1.746(0.365) | 0.071 |
| Lateral | λ_1_ | 0.477 | 1.169(0.302) | 0.218 | 0.738 | 1.139(0.280) | 0.143 | 0.689 | 0.978(0.188) | 0.105 |
|  | λ_2_ | 0.409 | 0.900(0.252) | 0.194 | 0.669 | 0.877(0.238) | 0.137 | 0.635 | 0.742(0.179) | 0.108 |
|  | λ_3_ | 0.244 | 0.631(0.201) | 0.175 | 0.502 | 0.611(0.186) | 0.131 | 0.570 | 0.520(0.152) | 0.100 |
|  | MD | 0.388 | 0.900(0.249) | 0.195 | 0.654 | 0.876(0.232) | 0.136 | 0.630 | 0.747(0.170) | 0.103 |
|  | FA | 0.514 | 0.339(0.079) | 0.055 | 0.616 | 0.342(0.080) | 0.049 | 0.566 | 0.367(0.089) | 0.059 |
|  | Mask Volume | 0.970 | 1.923(0.493) | 0.085 | 0.967 | 1.943(0.498) | 0.091 | 0.992 | 1.627(0.337) | 0.030 |
| Proximal | λ_1_ | 0.403 | 1.158(0.284) | 0.219 | 0.630 | 1.125(0.248) | 0.151 | 0.783 | 0.990(0.226) | 0.105 |
|  | λ_2_ | 0.337 | 0.901(0.245) | 0.199 | 0.567 | 0.876(0.224) | 0.147 | 0.808 | 0.757(0.227) | 0.100 |
|  | λ_3_ | 0.285 | 0.654(0.210) | 0.178 | 0.471 | 0.633(0.195) | 0.142 | 0.833 | 0.534(0.209) | 0.085 |
|  | MD | 0.343 | 0.905(0.244) | 0.198 | 0.561 | 0.878(0.220) | 0.146 | 0.808 | 0.760(0.219) | 0.096 |
|  | FA | 0.542 | 0.329(0.078) | 0.053 | 0.644 | 0.331(0.079) | 0.047 | 0.816 | 0.383(0.108) | 0.046 |
|  | Mask Volume | 0.970 | 2.868(0.701) | 0.120 | 0.968 | 2.889(0.714) | 0.128 | 0.980 | 2.394(0.483) | 0.068 |
| Distal | λ_1_ | 0.164 | 1.041(0.241) | 0.221 | 0.687 | 1.008(0.196) | 0.110 | 0.824 | 0.922(0.201) | 0.084 |
|  | λ_2_ | 0.292 | 0.767(0.201) | 0.169 | 0.739 | 0.744(0.178) | 0.091 | 0.813 | 0.676(0.186) | 0.080 |
|  | λ_3_ | 0.280 | 0.514(0.168) | 0.143 | 0.755 | 0.495(0.150) | 0.074 | 0.797 | 0.448(0.159) | 0.071 |
|  | MD | 0.220 | 0.774(0.200) | 0.177 | 0.724 | 0.749(0.171) | 0.090 | 0.813 | 0.682(0.180) | 0.078 |
|  | FA | 0.447 | 0.391(0.085) | 0.063 | 0.688 | 0.394(0.085) | 0.048 | 0.717 | 0.430(0.090) | 0.048 |
|  | Mask Volume | 0.966 | 2.588(0.776) | 0.143 | 0.964 | 2.616(0.787) | 0.150 | 0.985 | 2.106(0.420) | 0.052 |
