# Supplementary material for: Reliability and Repeatability of Diffusion Tensor Imaging in Healthy and Pathological Patellar Tendons
Source: J Orthop Res. 2026 Jan 29;44(2):e70156. doi: 10.1002/jor.70156 (PMC12853323; doi:10.1002/jor.70156)
Supplement: Supplementary file 5 — Table S3: Test‐retest repeatability intraclass correlation (ICC) results pooling all limbs and including limb type (healthy, tendinopathy, BPTB) as a fixed effect to model overall ICC measures and calculate confidence intervals (CIs) [ICC(CI)] for diffusion tensor imaging (DTI) diffusivities (λ1, λ2, and λ3), mean diffusivity (MD), fractional anisotropy (FA), and mask volume across tendon regions. [file JOR-44-0-s004.docx]

**Table S-3.** Test-retest repeatability intraclass correlation (ICC) results pooling all limbs and including limb type (healthy, tendinopathy, BPTB) as a fixed effect to model overall ICC measures and calculate confidence intervals (CIs) [ICC(CI)] for diffusion tensor imaging (DTI) diffusivities (λ_1_, λ_2,_ and λ_3_), mean diffusivity (MD), fractional anisotropy (FA), and mask volume across tendon regions. The identified pathological tendon outlier was excluded from this analysis.

| **Region** | **DTI Metric** | **All Tendons (N = 19)** |
| --- | --- | --- |
| Whole Tendon | λ_1_ | 0.645 (0.507, 0.936) |
|  | λ_2_ | 0.734 (0.569, 0.949) |
|  | λ_3_ | 0.747 (0.559, 0.956) |
|  | MD | 0.706 (0.535, 0.949) |
|  | FA | 0.824 (0.774, 0.956) |
|  | Mask Volume | 0.827 (0.801, 0.976) |
| Medial | λ_1_ | 0.809 (0.589, 0.907) |
|  | λ_2_ | 0.876 (0.687, 0.980) |
|  | λ_3_ | 0.903 (0.726, 0.984) |
|  | MD | 0.871 (0.666, 0.979) |
|  | FA | 0.905 (0.851, 0.975) |
|  | Mask Volume | 0.716 (0.682, 0.926) |
| Central | λ_1_ | 0.469 (0.346, 0.904) |
|  | λ_2_ | 0.610 (0.455, 0.929) |
|  | λ_3_ | 0.646 (0.497, 0.932) |
|  | MD | 0.568 (0.413, 0.928) |
|  | FA | 0.778 (0.724, 0.939) |
|  | Mask Volume | 0.814 (0.779, 0.971) |
| Lateral | λ_1_ | 0.606 (0.554, 0.916) |
|  | λ_2_ | 0.601 (0.535, 0.915) |
|  | λ_3_ | 0.513 (0.389, 0.905) |
|  | MD | 0.577 (0.503, 0.911) |
|  | FA | 0.577 (0.491, 0.898) |
|  | Mask Volume | 0.855 (0.834, 0.974) |
| Proximal | λ_1_ | 0.495 (0.313, 0.947) |
|  | λ_2_ | 0.584 (0.331, 0.958) |
|  | λ_3_ | 0.567 (0.251, 0.964) |
|  | MD | 0.545 (0.312, 0.957) |
|  | FA | 0.722 (0.583, 0.958) |
|  | Mask Volume | 0.880 (0.851, 0.975) |
| Distal | λ_1_ | 0.600 (0.525, 0.907) |
|  | λ_2_ | 0.677 (0.594, 0.929) |
|  | λ_3_ | 0.687 (0.584, 0.933) |
|  | MD | 0.651 (0.557, 0.924) |
|  | FA | 0.680 (0.591, 0.917) |
|  | Mask Volume | 0.725 (0.682, 0.976) |
