# Supplementary material for: Reliability and Repeatability of Diffusion Tensor Imaging in Healthy and Pathological Patellar Tendons
Source: J Orthop Res. 2026 Jan 29;44(2):e70156. doi: 10.1002/jor.70156 (PMC12853323; doi:10.1002/jor.70156)
Supplement: Supplementary file 6 — Table S4: Mean and standard deviation (SD) measures of diffusion tensor imaging (DTI) diffusivities (λ1, λ2, and λ3) [10−3mm2/s], mean diffusivity (MD) [10−3mm2/s], fractional anisotropy (FA), and mask volume [cm3] across regions within pathological and contralateral patellar tendons segmented by Rater 1 from the first scan session. [file JOR-44-0-s002.docx]

**Table S-4.** Mean and standard deviation (SD) measures of diffusion tensor imaging (DTI) diffusivities (λ_1_, λ_2,_ and λ_3_) [10^-3^mm^2^/s], mean diffusivity (MD) [10^-3^mm^2^/s], fractional anisotropy (FA), and mask volume [cm^3^] across regions within pathological and contralateral patellar tendons segmented by Rater 1 from the first scan session.

| **Tendon Region** | **DTI Metric** | **BPTB**  **[Mean(SD)]**  N = 5 | **Tendinopathy [Mean(SD)]**  N = 5 | **Contralateral [Mean(SD)]**  N = 10 |
| --- | --- | --- | --- | --- |
| Whole Tendon | λ_1_ | 1.181(0.251) | 0.990(0.085) | 0.922(0.170) |
|  | λ_2_ | 0.897(0.228) | 0.755(0.084) | 0.689(0.172) |
|  | λ_3_ | 0.633(0.207) | 0.536(0.076) | 0.469(0.161) |
|  | MD | 0.904(0.227) | 0.760(0.080) | 0.693(0.166) |
|  | FA | 0.369(0.010) | 0.353(0.050) | 0.412(0.103) |
|  | Mask Volume | 6.276(1.353) | 4.560(0.970) | 4.439(0.827) |
| Medial | λ_1_ | 1.033(0.209) | 1.129(0.087) | 1.014(0.229) |
|  | λ_2_ | 0.771(0.212) | 0.866(0.087) | 0.779(0.232) |
|  | λ_3_ | 0.545(0.209) | 0.622(0.107) | 0.532(0.223) |
|  | MD | 0.783(0.209) | 0.872(0.090) | 0.775(0.226) |
|  | FA | 0.410(0.110) | 0.356(0.079) | 0.411(0.105) |
|  | Mask Volume | 1.330(0.322) | 1.044(0.214) | 1.084(0.197) |
| Central | λ_1_ | 1.159(0.220) | 0.931(0.079) | 0.871(0.180) |
|  | λ_2_ | 0.884(0.197) | 0.693(0.082) | 0.639(0.180) |
|  | λ_3_ | 0.627(0.185) | 0.486(0.070) | 0.426(0.162) |
|  | MD | 0.890(0.197) | 0.703(0.076) | 0.645(0.173) |
|  | FA | 0.360(0.102) | 0.374(0.045) | 0.433(0.110) |
|  | Mask Volume | 2.723(0.594) | 1.825(0.407) | 1.748(0.337) |
| Lateral | λ_1_ | 1.292(0.337) | 0.990(0.115) | 0.938(0.165) |
|  | λ_2_ | 0.989(0.298) | 0.774(0.110) | 0.704(0.160) |
|  | λ_3_ | 0.691(0.254) | 0.554(0.085) | 0.491(0.149) |
|  | MD | 0.991(0.296) | 0.773(0.102) | 0.711(0.155) |
|  | FA | 0.360(0.109) | 0.319(0.042) | 0.381(0.104) |
|  | Mask Volume | 2.223(0.494) | 1.692(0.365) | 1.611(0.330) |
| Proximal | λ_1_ | 1.261(0.326) | 1.047(0.085) | 0.947(0.193) |
|  | λ_2_ | 0.994(0.293) | 0.815(0.085) | 0.720(0.203) |
|  | λ_3_ | 0.730(0.271) | 0.596(0.082) | 0.504(0.190) |
|  | MD | 0.995(0.297) | 0.819(0.082) | 0.724(0.194) |
|  | FA | 0.330(0.107) | 0.324(0.040) | 0.395(0.155) |
|  | Mask Volume | 3.329(0.577) | 2.458(0.565) | 2.389(0.468) |
| Distal | λ_1_ | 1.077(0.198) | 0.929(0.127) | 0.900(0.166) |
|  | λ_2_ | 0.776(0.199) | 0.690(0.112) | 0.657(0.155) |
|  | λ_3_ | 0.510(0.186) | 0.471(0.097) | 0.432(0.143) |
|  | MD | 0.788(0.191) | 0.697(0.110) | 0.663(0.152) |
|  | FA | 0.417(0.109) | 0.383(0.074) | 0.432(0.099) |
|  | Mask Volume | 3.001(0.807) | 2.140(0.433) | 2.090(0.401) |
